# Programmatic Adoption and Implementation of Video-Observed Therapy in Minnesota: Prospective Observational Cohort Study

**DOI:** 10.2196/38247

**Published:** 2022-08-05

**Authors:** Preetham Bachina, Christopher Kirk Lippincott, Allison Perry, Elizabeth Munk, Gina Maltas, Rebecca Bohr, Robert Bryan Rock, Maunank Shah

**Affiliations:** 1 Division of Infectious Diseases Johns Hopkins School of Medicine Baltimore, MD United States; 2 Division of Epidemiology Department of Population Health New York University School of Medicine New York, NY United States; 3 Hennepin County Public Health Minneapolis, MN United States; 4 Division of Infectious Disease Department of Medicine Hennepin Healthcare Minneapolis, MN United States

**Keywords:** video directly observed therapy, vDOT, mobile health, mHealth, tuberculosis, medication adherence, telemedicine, treatment, telehealth, observed therapy, COVID-19, primary outcome, treatment adherence, technology adoption, virtual health

## Abstract

**Background:**

In-person directly observed therapy (DOT) is standard of care for tuberculosis (TB) treatment adherence monitoring in the US, with increasing use of video-DOT (vDOT). In Minneapolis, vDOT became available in 2019.

**Objective:**

In this paper, we aimed to evaluate the use and effectiveness of vDOT in a program setting, including comparison of verified adherence among those receiving vDOT and in-person DOT. We also sought to understand the impact of COVID-19 on TB treatment adherence and technology adoption.

**Methods:**

We abstracted routinely collected data on individuals receiving therapy for TB in Minneapolis, MN, between September 2019 and June 2021. Our primary outcomes were to assess vDOT use and treatment adherence, defined as the proportion of prescribed doses (7 days per week) verified by observation (in person versus video-DOT), and to compare individuals receiving therapy in the pre–COVID-19 (before March 2020), and post–COVID-19 (after March 2020) periods; within the post–COVID-19 period, we evaluated early COVID-19 (March-August 2020), and intra–COVID-19 (after August 2020) periods.

**Results:**

Among 49 patients with TB (mean age 41, SD 19; n=27, 55% female and n=47, 96% non–US born), 18 (36.7%) received treatment during the post–COVID-19 period. Overall, verified adherence (proportion of observed doses) was significantly higher when using vDOT (mean 81%, SD 17.4) compared to in-person DOT (mean 54.5%, SD 10.9; *P*=.001). The adoption of vDOT increased significantly from 35% (11/31) of patients with TB in the pre–COVID-19 period to 67% (12/18) in the post–COVID-19 period (*P*=.04). Consequently, overall verified (ie, observed) adherence among all patients with TB in the clinic improved across the study periods (56%, 67%, and 79%, *P*=.001 for the pre–, early, and intra–COVID-19 periods, respectively).

**Conclusions:**

vDOT use increased after the COVID-19 period, was more effective than in-person DOT at verifying ingestion of prescribed treatment, and led to overall increased verified adherence in the clinic despite the onset of the COVID-19 pandemic.

## Introduction

Tuberculosis (TB) remains a leading cause of infectious disease death globally and a contributor to morbidity and mortality in the United States [1-3]. Adherence to a TB therapeutic regimen can be difficult, owing in part to its long treatment course over several months [1,4-6]. Incomplete treatment adherence can result in treatment failure, development of multidrug resistant strains, and poor clinical outcomes [7,8].

Directly observed therapy (DOT) has historically been regarded as the standard of care to document treatment adherence in most US public health TB clinics and involves a health care provider observing a patient take their TB medication [4,9-11]. It should be acknowledged that DOT is a multifaceted intervention that has heterogenous implementation globally, with mixed data on effectiveness [12-14]. Nonetheless, in 2016, the US Centers for Disease Control and Prevention, American Thoracic Society, and Infectious Diseases Society of America guideline update suggested using DOT over self-administered therapy for routine treatment of TB [4].

While DOT offers the ability to document medication ingestion and couple adherence interventions (eg, psychological support and case management), implementation may carry substantial inconvenience, cost, or stigma for patients and service providers [15-17]. Furthermore, although current US and international guidance advocate for daily, 7-day TB treatment regimens, logistical constraints result in only partial documentation of adherence—DOT is commonly implemented only during weekdays, with self-report on weekends (ie, nearly one-third of all prescribed doses) [4,16]. New strategies to document TB treatment adherence using digital adherence technologies may allow more comprehensive ascertainment of adherence estimates in a more patient-centered manner [18-20].

Recent World Health Organization guidance has suggested that video directly observed therapy (vDOT) may replace DOT when video communication technology is available and can be appropriately administered, and its use has increased in US settings [9,16]. vDOT uses computer and other mobile devices to either synchronously (real time) or asynchronously (recorded) monitor a patient taking their TB medication and promote treatment adherence remotely [16,21,22]. Other benefits include facilitating adherence monitoring 7 days per week, being less resource intensive, and allowing flexibility in the timing of medication use for patients [16,20-23].

Previous studies, including randomized trials, demonstrated either noninferiority of vDOT to DOT for verifying scheduled weekday doses, or found that a greater proportion of prescribed doses can be verified using vDOT under study conditions [18,24-27]. Our group has previously assessed vDOT implementation under routine programmatic circumstances in a large urban clinic in the United States after an initial pilot period, and similarly found that vDOT led to higher proportions of verified prescribed doses than in person [20]. However, there are limited data on vDOT effectiveness and technology adoption in programs without prior experience with the technology.

We sought to evaluate vDOT use and effectiveness in a clinic with no prior vDOT experience and to understand patterns of technology adoption since the onset of the COVID-19 pandemic. In the Hennepin County Minnesota Public Health Department, the standard of care for TB treatment monitoring before Sept 2019 involved in-person DOT, Monday to Friday; vDOT technology was subsequently made available for routine use at the discretion of the TB clinic. We assessed the initial implementation of vDOT into the clinic and characterized technology effectiveness and adoption over time through a prospective pragmatic implementation study, beginning in 2019 when vDOT became available.

## Methods

### Overview

We conducted a pragmatic, prospective observational cohort study of TB treatment monitoring measured by self-report, in-person DOT, and asynchronous vDOT under routine conditions at the Hennepin County Public Health Clinic’s Tuberculosis Program in Minneapolis Minnesota, a setting without prior vDOT use or experience [22].

### Ethical Considerations

Patients with signed disclosures and authorization for release of records in accordance with the Minnesota Health Records Act were included. Protocols were approved by the ethics committees at Johns Hopkins University, with reliance agreements established with Hennepin Healthcare Research Institute (IRB00174219).

### Study Population

We abstracted routinely collected clinical data from electronic medical records for patients receiving treatment for active TB, who had signed disclosures and authorization for release of records in accordance with the Minnesota Health Records Act, from Sept 2019 to March 2021, with treatment follow-up available until June 2021 [28]. We only included patients who were ≥18 years of age, as pediatric patients may have different considerations for using DOT and vDOT warranting a dedicated study [29]. In addition, we only included patients with ≥2 months of therapy remaining to ensure participants had sufficient follow-up time to measure adherence. vDOT (emocha Mobile health) became available for use within the Hennepin County Public Health Clinic TB program beginning Sept 2019 (Figure S1 in Multimedia Appendix 1).

### Tuberculosis Care

As part of routine care, the TB program individualizes the modality of TB treatment monitoring (ie, self-report, vDOT, and in-person DOT) using locally developed protocols and in accordance with Hennepin County Public Health Clinic guidelines, following a shared decision-making paradigm with patients [30]. Local protocols excluded vDOT initiation in patients with current positive sputum acid-fast bacillus (AFB) smears; vDOT initiation was considered once patients were smear negative. There were no exclusion criteria for patients with drug-resistant TB or prior treatment adherence when determining modality for TB treatment monitoring. Providers and patients are allowed to switch from treatment modalities as deemed necessary based on individual circumstances.

Most patients with active TB were treated with standard therapy (rifampin, isoniazid, pyrazinamide, and ethambutol) 7 days per week. Routine treatment monitoring for in-person DOT included a health care worker observing treatment ingestion at the patient’s home or agreed location during weekdays (ie, Monday to Friday), with the exception of government holidays; other doses were self-administered, and adherence was determined by self-report.

Patients using vDOT were instructed to submit videos documenting ingestion of medications according to their prescribed schedule (ie, 7 days per week), and were given initial training into the vDOT software including demonstrations and instructions [31]. Patients received SMS reminders twice per day on days a video was expected, and the software allowed for secure chat between the patient and health care team in the case of questions or issues; the software was available in multiple languages. Videos were reviewed by either a nurse case manager or community health worker, typically the next business day. Patient inquiries were triaged and answered by either nurse case managers or community health workers during business hours.

### Statistical Analysis

The “reach” or use of vDOT was defined as the proportion of patients in whom vDOT was used for treatment monitoring. We calculated effectiveness based on the *verified adherence,* defined as the proportion of total prescribed doses that were verified by in-person DOT or vDOT. Unobserved doses were considered either missed or “self-administered” if reported to be taken by the patient and documented as self-reported adherence in clinical charts (ie, doses during the weekend, holidays, or other occasions during a period of in-person DOT monitoring) [18,20,24]. We assessed observation time periods as “in-person” or “vDOT” based on the scheduled modality for treatment monitoring. We used 2-sample, 2-tailed *t* tests and chi-square tests to quantify the differences in clinical and demographic characteristics comparing in-person DOT and vDOT, at an alpha of .05 to determine statistical significance. We assessed the association of potentially relevant clinical and demographic factors with the receipt of vDOT using a multivariable logistic regression; covariates were included in the model based on clinical relevance to the outcome of interest (age, sex, race, English proficiency, alcohol use, resistance, initial AFB smear status, site of TB, and COVID-19 period). To evaluate vDOT use over time due to increased experience with the tool and to assess the impact of COVID-19, we divided the observation period into approximately 6-month increments: September 2019-February 2020 (ie, pre–COVID-19 period), March 2020-August 2020 (early COVID-19 period), and September 2020-March 2021 (intra–COVID-19 period); periods after March 2020 were the considered post–COVID-19 period. All analyses were conducted in STATA 16 (StataCorp).

## Results

### Participant Characteristics

A total of 96 patients received treatment for active TB during the study period in the health department TB clinic, of which 49 (51%) signed disclosures allowing their charts to be abstracted for this study (n=31, 32% in the pre–COVID-19, n=11, 11% in the early COVID-19, and n=7, 7% in the intra–COVID-19 periods). Moreover, 96% (47/49) of the studied patients were non–US born, with the most commonly reported primary languages being English (28/49, 57%), Somali (11/49, 22%), Spanish (4/49, 8%), and Hmong (3/49, 6%; Table 1). Patients were classified as having pulmonary TB (n=20, 41%), extrapulmonary TB (n=22, 45%), or both (n=7, 14%; Table 1). Additionally, 7 (14%) patients had drug resistant disease, and 22 (45%) patients had AFB smear-positive disease at treatment onset (Table 1). The median treatment duration was 29.7 weeks (IQR 26-43), and it was longer in patients with exclusively pulmonary TB (median 38 weeks, IQR 29-66) compared to those with some extrapulmonary TB (median 27.5 weeks, IQR 26-39; *P*=.02). Additional patient characteristics are shown in Table 1.

**Table 1:** Patient characteristics.

Baseline characteristics^a^			All patients (n=49)		Any vDOT^b^ (n=23)		No vDOT^c^ (n=26)		*P* value
Age (years), mean (SD)^d^			40.9 (19)		35.0 (17)		46.2 (19)		.04
**Patient sex, n (%)**									.01
	Male	22 (45)		6 (26)		16 (62)			
	Female	27 (55)		17 (74)		10 (38)			
**Non–US born, n (%)**									.93
	No	2 (4)		1 (4)		1 (4)			
	Yes	47 (96)		22 (96)		25 (96)			
**Ethnicity, n (%)**									.99
	Not Hispanic	43 (88)		20 (87)		23 (88)			
	Hispanic	4 (8)		2 (9)		2 (8)			
	Unknown or not reported	2 (4)		1 (4)		1 (4)			
**Race, n (%)**									.42
	Asian	12 (25)		8 (35)		4 (15)			
	Black or African American	29 (59)		11 (48)		18 (69)			
	White	2 (4)		1 (4)		1 (4)			
	Unknown or not reported	6 (12)		3 (13)		3 (12)			
**English proficient, n (%)**									.62
	No	21 (43)		9 (39)		12 (46)			
	Yes	28 (57)		14 (61)		14 (54)			
**Experiencing homelessness, n (%)**									.34
	No	48 (98)		23 (100)		25 (96)			
	Yes	1 (2)		0 (0)		1 (4)			
**HIV infected, n (%)**									.09
	No	46 (94)		23 (10)		23 (88)			
	Yes	3 (6)		0 (0)		3 (12)			
**Any alcohol use, n (%)**									.04
	No	37 (76)		15 (65)		22 (85)			
	Yes	4 (8)		1 (5)		3 (11)			
	Unknown or not reported	8 (16)		7 (30)		1 (4)			
**TB^e^ drug resistance,^f^ n (%)**									.53
	No	35 (72)		17 (74)		18 (69)			
	Yes	7 (14)		2 (9)		5 (19)			
	Unknown or not reported	7 (14)		4 (17)		3 (12)			
**Initial AFB^g^ smear, n (%)**									.98
	No	25 (51)		12 (52)		13 (50)			
	Yes	22 (45)		10 (44)		12 (46)			
	Unknown or not reported	2 (4)		1 (4)		1 (4)			
**Site of TB**									.27
	PTB^h^	20 (41)		8 (35)		12 (46)			
	EPTB^i^	22 (45)		13 (56)		9 (35)			
	PTB and EPTB	7 (14)		2 (9)		5 (19)			

^a^There were no significant differences in age, ethnicity, birth country, English proficiency, employment, homelessness, HIV, drug resistance, initial acid-fast bacillus smear status, or site of tuberculosis (TB) across the study periods. There were significantly more females with TB in the post–COVID-19 period (14/18, 78%) compared with the pre–COVID-19 period (13/31, 42%; *P*=.02).

^b^vDOT: video directly observed therapy.

^c^No vDOT represents a combination of patients with self-administered and in-person directly observed therapy.

^d^Age as of TB treatment start date.

^e^TB: tuberculosis.

^f^No patients at the clinic were treated with injectable medications during the study period.

^g^AFB: acid-fast bacillus.

^h^PTB: pulmonary tuberculosis.

^i^EPTB: extrapulmonary tuberculosis.

### Reach of vDOT Compared to In-Person DOT

All patients had treatment monitored using in-person DOT, video-DOT, or some combination. vDOT was used for some portion of care in 47% (23/49) of patients, while the remainder (26/49, 53%) were monitored exclusively through in-person DOT (with self-administration during weekends, holidays, and per-clinic discretion; Table 1).

Overall, there was a trend toward increasing vDOT use when comparing each 6-month period, with 35% (11/31) using vDOT in the first 6 months after the technology became available (ie, pre–COVID-19), 64% (7/11) in the second 6 month (early COVID-19 period), and 71% (5/7) in the final (intra–COVID-19) period (ie, 1 year after vDOT became available; *P*=.10; Table 2). When comparing the pre–COVID-19 period to after the onset of COVID-19 (ie, early and intra–COVID-19 periods combined), significantly more patients used vDOT in the post–COVID-19 period (12/18, 67%) compared to the pre–COVID-19 period (11/31, 35%; *P*=.04). Among individuals initiating therapy after vDOT was available, the median time to start vDOT relative to TB treatment initiation date was 7 days (IQR 0-78). In-person DOT was initiated at the same time as treatment in the clinic (median 0 days, IQR 0-26). There was no difference in the overall treatment duration among those receiving vDOT (median 29.7 weeks, IQR 26-39.4) and those who did not receive vDOT (median 31, IQR 26.1-52; *P*=.67).

**Table 2:** Primary outcomes by study period.

Variable			Overall (n=49)		Study period 1^a^ (n=31)		Study period 2^a^ (n=11)		Study period 3^a^ (n=7)		*P* value^b^
vDOT^c^ use, n (%)			23 (47)		11 (35)		7 (64)		5 (71)		.10
**Verified adherence (%), mean (SD)^d^**											
	vDOT^e^	81 (17.4)^f^		76.1 (19.9)		81.9 (16.6)		90.7 (9.0)		.31	
	In-person DOT^e,g^	54.5 (10.9)^f^		54.6 (9.8)		47.7 (13.3)		65.4 (6.7)		.03	
	Overall (irrespective of monitoring modality)	61.7 (16.6)		56.1 (10.0)		66.6 (24.2)		79 (13.3)		.001	
**Self-administered therapy (%), mean (SD)^h^**											
	During vDOT	10.8 (12.9)		15.6 (15.6)		6.6 (9.3)		6.1 (7.5)		.24	
	During in-person DOT	44.1 (10.9)		44.2 (10.1)		49.1 (14.0)		34.6 (6.7)		.11	
	Overall (irrespective of monitoring modality)	35.6 (17.2)		41.9 (10.2)		28.6 (24.5)		18.8 (14.9)		<.001	

^a^Study period 1 is defined as the first 6 months of the study period from September 2019 to February 2020. Study period 2 is defined as the second 6 months of the study period from March 2020 to August 2020. Study period 3 is defined as the final study period from September 2020 to March 2021.

^b^*P* values represent comparisons across study periods (by row).

^c^vDOT: video directly observed therapy.

^d^Verified adherence is defined as doses that were observed by either in-person or vDOT divided by the total number of prescribed doses.

^e^Comparing verified adherence between those receiving vDOT and in-person DOT, adherence was higher in all study periods when using vDOT (*P*<.001, *P*=.001, *P*=.002, for periods 1, 2, and 3, respectively).

^f^The overall verified adherence from vDOT (median 86%, IQR 71-99), was significantly greater than the overall verified adherence from in-person DOT (median 57%, IQR 47-63, *P*=.001).

^g^DOT: directly observed therapy.

^h^A greater proportion of doses was self-administered when using in-person DOT compared to vDOT overall and in each study period (*P*<.001 for all comparisons).

In univariate analysis (Table 1), mean age of individuals receiving vDOT was lower (35) compared to those not receiving vDOT (46; *P*=.04); a larger proportion of women (17/27, 63%) received vDOT compared to men (6/22, 27%; *P*=.01; Table 1). However, in multivariate analysis adjusting for other covariates, the adjusted odds ratios (AORs) revealed that neither sex (AOR 0.23, 95% CI 0.29-1.83) nor age category (AOR 0.27, 95% CI 0.03-2.5; AOR 3.0, 95% CI 0.02-455; and AOR 0.06, 95% CI 0.01-2.4 for individuals 30-50, 50-65, and >65 years old compared to those <30 years old, respectively) was associated with vDOT use. There was a trend toward increased vDOT use after the onset of COVID-19 (AOR 10.1, 95% CI 0.57-176; *P*=.11), but it was not statistically significant; no other clinical or demographic features, including race, English proficiency, alcohol use, site of TB disease, initial smear status, or drug resistance, were found to be associated with vDOT use. All patients successfully completed treatment irrespective of adherence monitoring modality (or were censored at the end of the study period).

### Effectiveness of vDOT Compared to In-Person DOT

Overall, the mean *verified adherence* (proportion of prescribed doses verified through observation) was significantly higher when using vDOT (mean 81%, SD 17.4; median 86%, IQR 71-99), compared to in-person DOT (mean 54.5%, SD 10.9; median 57%, IQR 47-63; *P*=.001; Table 2).

These findings were driven by the high proportion of “self-administered” doses when using in-person DOT (mean 44.1%, SD 10.9; median 41%, IQR 36-47) compared to periods when patients were using vDOT (mean 10.8%, SD 12.9; median 5%, IQR 0-16; *P*<.001). By contrast, few doses were documented as missed (median <1%, IQR 0-1.2) when using in-person DOT; overall, among prescribed doses (7 days per week), a median of 1% (IQR 0-11) was documented as missed when using vDOT (*P*=.11).

### Effectiveness of vDOT Compared to In-Person DOT by Study Period

We found a trend toward greater verified adherence using vDOT over the time period of implementation, but it was not statistically significant (mean 76%, 82%, and 91%, in the pre–COVID-19, early COVID-19, and intra–COVID-19 study periods, respectively; *P*=.31; Table 2). In all time periods, adherence was higher when using vDOT compared to in-person DOT (*P*<.01 for each period; Table 2).

As a result of an increasing proportion of patients in whom vDOT was used for adherence monitoring, we found that overall adherence increased in the population across successive periods of observation (mean 56%, 67%, and 79% for each study period, respectively; *P*=.001; Table 2). We also found that the overall proportion of treatment doses documented as self-administered declined across study periods (42%, 29%, and 19%, respectively; *P*<.001; Table 2), which is attributable to the increasing vDOT use.

## Discussion

### Principal Results

Paradigms for documenting TB treatment adherence are evolving given the logistical constraints with in-person DOT since the onset of the COVID-19 pandemic and with the prioritization of daily TB therapy 7 days per week [4,9]. Several clinical trials have reported that video-observed therapy is effective at documenting TB treatment under study conditions, but data to inform programmatic implementation are limited [25,27]. We previously reported that vDOT use was high under routine conditions in a clinic with established prior vDOT experience. In this prospective cohort study, at a vDOT naïve clinic (ie, no prior experience) in Minnesota, we found progressively increasing adoption of vDOT for patients with active TB disease [20]. Unsurprisingly, initial use of vDOT was slow (n=11, 35% of patients; Table 2); however, 1 year after vDOT availability, we found the TB program used vDOT preferentially in nearly three-quarters of patients. The shift toward monitoring treatment for all prescribed doses has clinical implications; vDOT was more effective than in-person DOT for verifying ingestion of prescribed TB treatment, allowing median documentation of adherence for 86% of the prescribed doses; by contrast, median in-person DOT verified adherence was 57%, owing to a large proportion of self-reported doses (Table 2). These data are consistent with findings in other study conditions [18,20,24,25]. With increasing vDOT use over time, we consequently found that the overall verified adherence among all patients in the clinic increased from 56% in the first 6 months of vDOT availability (pre–COVID-19 period) to 79% in the last 6 months of the study, despite programmatic disruptions and diminished staff during the COVID-19 pandemic (Table 2).

Several factors likely influenced our results and the observed “digital transformation” of health care. The adoption of vDOT in the Hennepin County Public Health Clinic TB program follows the “technology-push” paradigm, in which health care workers are in the process of implementing a tool foreign to them [32]. Commonly, the novelty of new tools and disruption to well-established routines often lead to lower initial use, as seen in our study [32]. Furthermore, health care providers’ perception of telemedicine may be negatively influenced by concerns surrounding telemedicine project funding, ease of use, and patient preferences [33].

The onset of the COVID-19 pandemic in March 2020 significantly impacted the TB clinic as well. The program staff of 4 full-time outreach Community Health Workers was reduced to 1.5 to 2 full-time equivalent workers at various times throughout the pandemic. Consequently, the reduction of in-person services caused by the COVID-19 pandemic likely accelerated the adoption of vDOT, along with growing comfort and experience with the technology. More generally, expansion of telemedicine and digitally enabled communication during the pandemic may also have contributed to greater acceptance of using digital adherence technologies among health care providers and patients [33,34].

In addition to these reasons, the continued use of vDOT technology over the course of the study period may also be partially due to the significantly larger proportion of prescribed doses that were verifiable using vDOT compared to in-person DOT. These findings are largely attributable to the reduction in the number of self-administered doses over weekends and holidays offered by vDOT compared to in-person DOT and is consistent with findings from other programmatic studies. Additionally, prior concerns within the Hennepin County TB program regarding costs and lost revenue associated with reimbursements were mitigated by the program’s ability to bill for vDOT visits based on local practices.

### Limitations

Our study has several limitations. Due to local regulatory requirements, we were only able to abstract data from patients who had signed a disclosure and authorization for release of information; alternatively, this patient decision was independent of our specific study, and is unlikely to have led to significant selection biases as related to choice of treatment monitoring modality or adherence. We also were unable to differentiate the impact of COVID-19 (eg, staffing changes and lockdowns) and the natural process of technology adoption as staff became more familiar and comfortable with the platform. Our study setting implemented vDOT 7 days per week to allow the assessment of adherence to daily prescribed therapy; our study results may not be generalizable to clinics that prescribe treatment according to alternative dosing schedules (eg, 5 days per week or thrice weekly).

### Comparison With Prior Works

While self-report or pill counts, in-person DOT, vDOT, and other digital technologies (eg, smart pill boxes) each provide a different level of certainty related to medication ingestion, they each broadly provide a measure of adherence. Our results add to the growing literature on the feasibility, acceptability, and effectiveness of video-DOT [18,20,24,25] for adherence measurements, but they also raise important considerations for the reevaluation of current paradigms in documenting TB treatment adherence. Recently, a large randomized trial found that electronic DOT was noninferior to in-person DOT at monitoring “scheduled” doses (Monday-Friday) but did not report adherence to all prescribed doses [27]. Our study highlights the limitations of this approach. Existing protocols for determining adherence used by many TB clinics offers incomplete assessments of true adherence by monitoring only a fraction of prescribed doses; for example, achieving 80% documentation of Monday-Friday doses (with limited certainty of ingestion of prescribed weekend doses) represents documentation of only 57% of prescribed doses. We acknowledge that there are currently limited data on the optimal thresholds for determining the proportion of prescribed treatment that should be verified. Moreover, correlation of treatment verification and specifically added weekend treatment verification with clinical outcomes is also limited. Nonetheless, our results show that adoption of vDOT in a large urban clinic allowed verification of true adherence to above 80%, including weekend doses, and offered more comprehensive categorization of all treatment doses. Previously, we have also found that vDOT may reduce stigma and increase logistical convenience, while also allowing TB programs to reduce costs and reallocate resources more efficiently [18]. Notably, treatment adherence improved during the course of the study despite reductions in staff.

### Conclusions

Our results should be interpreted in the context of individualized decision-making as is advocated by current guidelines. Some individuals continued to receive in-person DOT based on tailored individual considerations; while our prior work has suggested that older age may be associated with a lower likelihood of initiating vDOT in other settings, we did not find specific clinical or demographic factors associated with vDOT selection in this clinic [24]. We also note that provisions for adherence support (eg, psychological support, nursing support, and other incentives and enablers) were made in conjunction with decisions on deciding treatment modality for monitoring; in this manner, adherence support interventions should be viewed adjunctively and are not synonymous with DOT or vDOT. Other elements of adherence support built into the chosen vDOT system included electronic reminders, secure chat, and ability to document symptoms and side effects, which may have also impacted the results.

## Appendix

Multimedia Appendix 1 Description of emocha platform for video-observed therapy: (a) Video directly observed therapy (DOT) application schematic; (b) Video DOT application user interface.

## Abbreviations

AFB: acid-fast bacillus
AOR: adjusted odds ratio
DOT: directly observed therapy
TB: tuberculosis
vDOT: video directly observed therapy
