# Supplementary material for: Programmatic Adoption and Implementation of Video-Observed Therapy in Minnesota: Prospective Observational Cohort Study
Source: JMIR Form Res. 2022 Aug 5;6(8):e38247. doi: 10.2196/38247 (PMC9359306; doi:10.2196/38247)
Supplement: Multimedia Appendix 1 [file formative_v6i8e38247_app1.docx]

## Multimedia Appendix 1

Figure S1 Description of emocha Platform for video-observed therapy: a) Video DOT application schematic b) Video DOT Application User Interface

Patients downloaded the emocha application with the assistance of clinic staff. The application is HIPAA compliant, and available in multiple languages. The application delivers SMS reminders to the patient to take their medication twice per day on days of scheduled dosages. Patients document treatment adherence through the application which takes a secure video, which is transmitted to a cloud server when internet connectivity is present. If offline, the application stores the video and uploads whenever connectivity is achieved. All videos are date and time-stamped at the time of recording. The application additionally prompts users for daily side-effect monitoring, and offers live-stream visits along with secure text chat. The health care worker dashboard allows review of the videos, and documentation of medication administration, and provides reports.


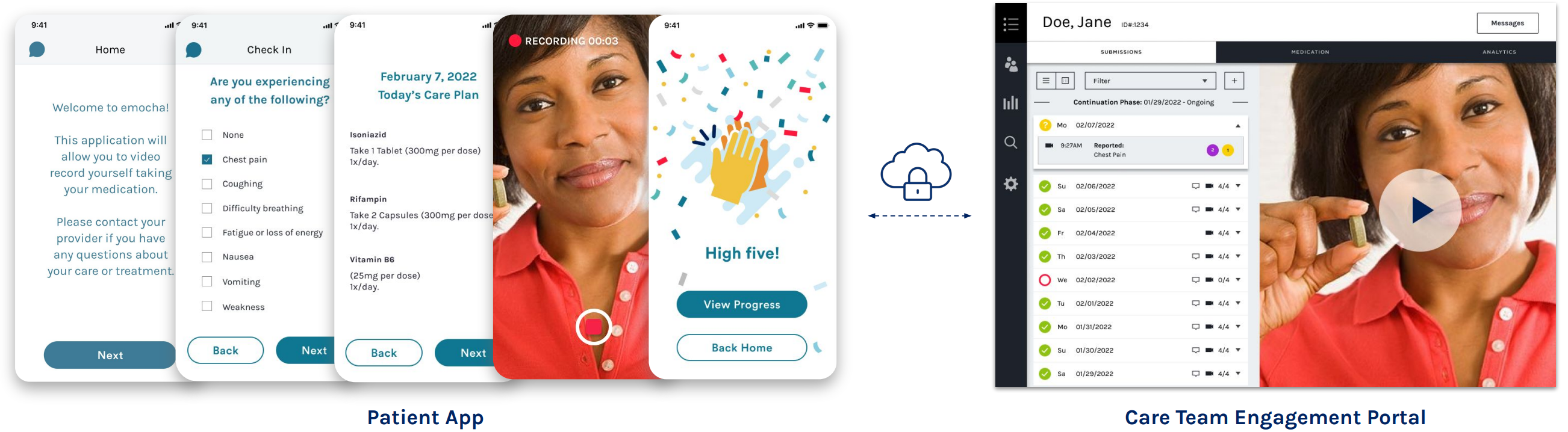


Figure S1b. Video DOT Application User Interface


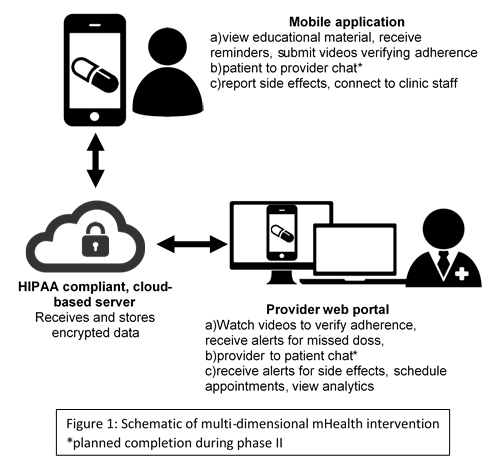


Figure S1a. Video DOT application schematic
